# Virology in Peril and the Greater Risk To Science

**DOI:** 10.1128/mbio.03339-22

**Published:** 2022-12-15

**Authors:** Michael J. Imperiale, Arturo Casadevall, Felicia D. Goodrum, Stacey Schultz-Cherry

**Affiliations:** a Department of Microbiology and Immunology, University of Michigan, Ann Arbor, Michigan, USA; b Department of Microbiology and Immunology, Bloomberg School of Public Health, Johns Hopkins University, Baltimore, Maryland, USA; c Department of Immunobiology, University of Arizona, Tucson, Arizona, USA; d Department of Infectious Diseases, St. Jude Children’s Research Hospital, Memphis, Tennessee, USA

## EDITORIAL

Late in 2019, a new zoonotic viral pathogen entered the human population: severe acute respiratory syndrome coronavirus 2 (SARS-CoV-2). It spread rapidly and relentlessly, inflicting great harm. Governments were unprepared to deal with it. Health care workers had to learn how to treat patients on the fly, devoting countless hours to care and putting themselves at risk. Schools and businesses closed, and most of the world found itself under lockdowns and other types of restrictions. Uncertainty loomed. How many people would get sick and die? What would the effects be on the economy? How far behind would our children fall in their education and social skill development? How long would it take to find a cure or a vaccine?

Fortunately, with respect to the last question, the scientific research enterprise was up to the task. Scientists who had been studying coronaviruses quickly redoubled their efforts, and hundreds of other virologists spun up efforts to understand this virus. Wise investments in basic research over prior decades meant that there was a wealth of knowledge on the shelf that could be used to develop vaccines rapidly (1). Companies that were developing new technologies accelerated their efforts to produce effective vaccines. Within a year, an amazing time frame that we think few scientists would have predicted, we began to immunize the population and drive down the incidence of serious illness and death. Parallel efforts led to the rapid development of monoclonal antibody therapies and small-molecule antiviral agents. Rapid diagnostic tests were developed, and we went from relying on relatively slow nucleic acid amplification methods that took days in the early days of the pandemic to home tests that provide a result in minutes. Virologists and evolutionary scientists tracked the evolution of the virus and rapidly provided information on the emergence of new variants, creating a viral forecast mechanism that has provided almost real-time information on the changing pandemic. This has all happened in less than 3 years and resulted in a great reduction in mortality. As such, we should be celebrating the virology community and the allied scientific communities.

Remarkably, despite the fact that the virology community made critical contributions to a remarkably successful response to the CoV disease 2019 (COVID-19) pandemic, it finds itself under attack. The bulk of this attack comes under the guise of concern about gain-of-function (GOF) research and persistent concerns about the origin of SARS-CoV-2. What began 10 years ago as a civil, scholarly debate over the experiments aimed at addressing whether highly pathogenic avian H5N1 influenza could become transmissible from human to human has devolved to include almost all experiments in which respiratory pathogens are genetically manipulated. While much of the conversation remains courteous and thoughtful, an ever-increasing fraction has become a negative, knee-jerk reaction. All virological research is being viewed with suspicion, with the criticisms often playing out in social, online, and traditional news media environments.

This type of behavior is not going to serve society well in the long run. The perception that virologists are not paying attention to the possible risks of their research is simply wrong and harmful. Virologists have expert knowledge of viruses and the diseases that they cause and are keenly aware of the need for strict biosafety and biosecurity practices. It is harmful to the virology community because it mischaracterizes their intentions, which are to protect lives from infectious disease. It is harmful to the public because it further diminishes their faith in research at a time when there is already significant distrust and disbelief, which is further fueled by misinformation being disseminated at a dizzying pace under the guise of science. If we cannot trust and support the scientists who strive each day to help us be better prepared for the next outbreak or pandemic, we surrender our future to the threat from nature, and we argue that we are doomed to have pathogens control us, rather than vice versa.

There are solutions to this problem. First, we call on the virology community to engender trust in what we do actively and enthusiastically. Not only do we have a responsibility to perform life-saving research, but we need to ensure that, if we study pathogens that do carry a significant level of risk, any given experiment (i) has benefits that are clearly delineated from the outset, (ii) is carefully vetted, and (iii) is carried out with the highest level of safety in mind. On top of that, we need to carry that message to the public in an understandable manner and in a way that is not condescending. We should clearly delineate the benefits of the research that we perform, including explaining why GOF is the preferred approach to reaping those benefits in those cases where it is. Second, we call on the community that has been advocating for a stop to all gain-of-function research to ask itself similar questions. We believe that the benefits of the research are sometimes forgotten or, at the least, downplayed by some of these individuals. Third, it is possible to analyze the types of experiments that should be done and not done based on a framework of need to know and risk, as we proposed in prior essays (2, 3). Finally, we call on both sides to please focus on the issues and stop the name-calling.

Today, virology is in the crosshairs of debate and criticism. However, other disciplines might soon find themselves under similar scrutiny, since not all dangerous pathogenic microbes are viruses. It is worthwhile to note that the Black Death, which is arguably the greatest recorded infectious disease calamity in human history, was caused by a bacterium and that today we are living through catastrophic declines in amphibians, bats, and salamanders caused by fungal diseases. Furthermore, entire disciplines, such as synthetic biology, a field that might bring revolutionary technologies for human good, might soon come under similar scrutiny. Hence, the situation in which virology finds itself today may be a forerunner for similar struggles that may ultimately involve all of biology.

There must be a path in which everyone involved can work together to develop a scheme for ensuring that research that addresses important societal problems can move forward. Might there be some experiments that address questions that are more purely academic in nature than practical? Most definitely yes. Such experiments should have a lower level of risk tolerance, and we need to admit that. Conversely, we must acknowledge that not every gain-of-function experiment carries the risk of global catastrophe. Two of us (A. Casadevall and M. Imperiale) served for 9 years on the National Science Advisory Board for Biosecurity and have remained engaged in the topic through academic contributions. It is our collective sense that most of the individuals involved in these debates are reasonable and understand the nuances. Unfortunately, as often happens in public debates involving issues of consequence, a much smaller number of people at the far extremes seem to have the loudest voices and the attention of the public via the media sources noted above. We call on the people in the middle to have a serious dialog and to come to a consensus on the best path forward. We ask our fellow virologists, scientific colleagues, and friends who are reading this editorial to step up and lead these efforts; we owe it to ourselves and the public as responsible scientists and members of society. We call on our colleagues from all scientific disciplines to become informed on the controversies involving virology research and to use their good offices to inform the public about the benefits of a healthy scientific enterprise for the well-being and future of humanity. All scientists and the public at large have a stake in the outcome of the current controversy. If our message is not thoughtful and targeted, we run the risk of others making decisions for us. There could hardly be a worse outcome as we emerge from the COVID-19 pandemic with an increased appreciation of the infectious disease risks that nature has in store for us. This outcome will leave us ill-prepared to respond to the next pandemic threat.

## References

[1] Casadevall A. 2021. The mRNA vaccine revolution is the dividend from decades of basic science research. J Clin Invest 131:e153721. 34558420 10.1172/JCI153721 PMC8483746

[2] Imperiale MJ, Casadevall A. 2018. A new approach to evaluating the risk-benefit equation for dual-use and gain-of-function research of concern. Front Bioeng Biotechnol 6:21. doi:10.3389/fbioe.2018.00021. 29568736 PMC5853790

[3] Imperiale MJ, Casadevall A. 2015. A new synthesis for dual use research of concern. PLoS Med 12:e1001813. doi:10.1371/journal.pmed.1001813. 25874461 PMC4397073

